# Management of Burn Scars: A Five-Year Retrospective Study

**DOI:** 10.7759/cureus.31448

**Published:** 2022-11-13

**Authors:** Raymond Challita, Nagham Bazzi, Elie Fazaa, Deoda Maassarani, Toni Habib, Mariam Bazzi, George Ghanime, Ziad Sleiman

**Affiliations:** 1 Plastic and Reconstructive Surgery, Faculty of Medicine, Lebanese University, Beirut, LBN; 2 Medicine, Lebanese University, Beirut, LBN; 3 Plastic Surgery, Geitaoui Hospital, Beirut, LBN; 4 Plastic Surgery, Lebanese Hospital Geitaoui University Medical Center (UMC), Aschrafieh, LBN; 5 Faculty of Medicine, Lebanese University, Beirut, LBN; 6 Medicine, Saint Joseph University of Beirut (USJ), Beirut, LBN; 7 Plastic and Reconstructive Surgery, Lebanese Hospital Geitaoui University Medical Center (UMC), Achrafieh, LBN; 8 Plastic and Reconstructive Surgery, Lebanese University, Beirut, LBN; 9 Surgery, Lebanese Hospital Geitaoui University Medical Center (UMC), Achrafieh, LBN

**Keywords:** plastic and reconstructive surgery, keloid scar, burn reconstruction, electrical burn, burn injury reparative medicine treatments

## Abstract

Introduction

Despite the heightened interest in the management and prevention of burn scars, only a few articles have been published that assess the risk factors for the development of burn scars. The relationship between admission to the burn unit and the need for reconstructive surgery, the effect of the burn area on the number of further surgeries needed, and the adverse event of the technique used in the reconstructive surgery is not widely explored in the literature. These unmet challenges are crucial for a standardized consensus about burn scar management.

Methods

A retrospective study of patients admitted for burn reconstructive surgeries was conducted. A total of 100 patients (mean age: 29 years old) were included in this study. Data were retrospectively collected by reviewing the patients' charts. Data were analyzed using the SPSS software, version 25.00 (SPSS Inc., Chicago, IL).

Results

The most common surgery performed was a release contracture with skin grafting (n = 93.93%). No significant difference was reported between the patient's age and the total number of surgeries. A significant difference was noted between the different techniques used and the total number of surgeries. Patients with release contracture surgery had higher scores of satisfaction and better functional outcome.

Conclusion

The most common surgery performed for scar treatment was contracture release coupled with skin grafting. The most common cause of burn in Lebanon was flame, and the most commonly affected anatomical area was the upper limb. Further studies recruiting patients from all over Lebanon and assessing their characteristics are now warranted.

## Introduction

The consequences of wound repair are not always discretional [1,2]. Scarring is a major complication that occurs in 100 million patients each year [1,2]. The latter can be psychologically devastating and stigmatizing, which affects the patient’s quality of life [2]. Scarring ranges from mature lines to abnormally raised hypertrophic scars and keloids [3]. The latter is very common occurring in 70% of patients [4]. Traumatic and iatrogenic keloids are linear, whereas burn keloids are diffuse and widespread on the skin [3].

Many invasive and noninvasive options are available for scar management. Depending on the scar type, the treatment consists of pressure garments, splinting, silicones, laser therapy, massage, corticosteroids, or reconstructive surgery [2,5]. Reconstructive surgery plays a crucial role in the treatment of scarring post burns, and 91% of patients receiving routine surgical procedures appreciate scar improvement [3]. Although the optimal treatment for scars is prevention, reconstructive surgery can improve the aesthetic appearance and restore normal function in 12.7% of patients admitted to burn centers [6].

The improvement in acute burn care has led to decreased mortality among burn patients. Various published articles talked about the procedures for burn reconstruction; however, few articles addressed the epidemiological criteria and characteristics of these patients.

Despite the heightened interest in burn scar management and prevention, there is little knowledge and scarce articles assessing the risk factors for burn scars, the relation between admission to a burn unit and the need for reconstructive surgery, the effect of burn area on a number of further surgeries needed, and the adverse event of the technique used in the reconstructive surgery. These unmet challenges are crucial for a standardized consensus about burn scar management. This study discusses the association between patient general characteristics and the need for reconstructive surgery.

## Materials and methods

A retrospective study was conducted and included patients admitted for burn reconstructive surgeries at the Lebanese Hospital Geaitoui University Medical Center from 2014 to 2019.

Ethical considerations

The protocol was approved by an independent Institutional Review Board (IRB) at the Lebanese Hospital Geaitoui University Medical Center. Standard protocols regarding anonymity and confidentiality were followed to guarantee the credibility of the study.

Participants and data collection

Participants included were (a) Lebanese and (b) patients admitted for burn reconstructive surgery after initial wound closure. Patients who died and those who did not comply with the inclusion criteria were excluded from the study.

Data was collected using the Google platform. Information regarding age, gender, social status, smoking status, surgery date, length of hospital stay, burn mechanism, total body surface area (TBSA), previous admission to burn unit, area of the burn, the technique involved, anatomical area of the present surgery, antibiotherapy postoperatively, complications postoperatively, the need for further surgeries, patient satisfaction, number of previous surgeries with technique involved and anatomical area, aesthetic outcome, and functional status were obtained. The need for further surgeries was assessed by two plastic surgeons at six months postoperatively. Patient satisfaction was measured using a written scale (the Likert scale), graded over 10, and comparing pre- and postoperative outcomes. The aesthetic outcome was based on the shape of the scar, whether it is flat, hypertrophic, or keloid scarring. Functional status improvement was assessed for patients with functional impairment preoperatively. The level of improvement was identified by the patient as excellent, good, or no improvement.

Data analysis

Data was exported from the electronic platform to the SPSS program, version 23 (IBM Corp., Armonk, NY). For descriptive analysis, means and standard deviation were used for quantitative variables, while the frequency and percentage were used for qualitative variables (dichotomous or multinomial). The bivariate analysis included a comparison of patients who needed further surgery versus those who did not; the McNemar and Chi-square tests were used to compare the two values. In addition, a paired t-test comparison was conducted for quantitative variables. Moreover, Pearson chi-squared test was used to compare qualitative variables, and the student's T-test was used for quantitative variables.

## Results

A total of 100 patients, 43 females (43%) and 57 males (57%), were included in this study. All patients meeting the inclusion criteria were included in the study. The mean age of the total patients was 29.23 years old. Operated anatomical areas were distributed accordingly: head and neck (n = 20, 20%), torso (n = 15, 15%), upper limb (n = 63, 63%), and lower limbs (n = 33, 33%). The most common surgery performed was release contracture with skin grafting (n = 93, 93%), local flaps (n = 17, 17%), distant flaps (n = 3, 3%), or acellular dermal matrixes (n = 1, 1%). Lipofilling, debridement, or removal of foreign material was carried out in three patients (3%). These procedures were either isolated or combined together. Burn mechanism distribution was described in Table 1.

**Table 1:** Burn mechanism distribution among patients

Burn mechanism	Frequency (percentage)
Chemical burn	1 (1%)
Contact burn	4 (4.3%)
Electric burn	15 (16.1%)
Flame burn	34 (36.6%)
Other (Frost bite, friction, radiation)	9 (9.7%)
Scald burn	30 (32.2%)
Total	93 (100%)

Patient characteristics and the total number of surgeries

No significant difference was seen in patients' age and the total number of surgeries. No significant difference was noted among patients' gender, marital status, smoking status, cause of the burn, and the anatomical area burned. The majority of patients with TBSA burn percentages greater than 30% and those who were previously admitted to the burn unit received multiple surgeries (Table 2).

**Table 2:** Patients' general characteristics and surgical history

Number of previous surgery (frequency)		1	2	3	4	P-values
Age range (years)	0-20	12	7	2	5	0.761
	21-40	4	1	2	1	
	41-60	3	1	0	2	
	More than 60	4	0	0	2	
Gender	Female	9	4	2	7	0.714
	Male	14	5	2	3	
Marital status	Married	7	1	2	4	0.706
	Single	16	8	2	6	
Smoking status	Yes	5	1	1	2	0.957
	No	18	8	3	8	
Total body surface area (TBSA) “burn percentage”	0%-10%	9	5	1	5	0.695
	11%-20%	6	0	1	1	
	21%-30%	1	1	0	0	
	>30%	4	1	2	3	
Patient had been previously in burn unit	Not admitted	9	5	0	2	0.414
	Previously admitted to burn unit	14	4	4	8	
Cause of burn: “burn mechanism”	Chemical burn	1	0	0	0	0.86
	Contact burn	2	0	0	0	
	Electric burn	3	2	1	2	
	Flame burn	3	2	1	4	
	Other	5	1	1	0	
	Scald burn	8	4	1	2	
Area burned	Head and neck	5	1	1	5	0.18
	Upper limbs	14	7	4	5	
	Thorax and abdomen	6	2	2	2	
	Lower limbs	15	3	2	3	
Technique used at first surgery	Distant flap	2	0	0	0	0.046
	Z plasty local flap	3	3	2	0	
	Release contracture with a skin graft	16	4	12	8	
	Other	5	3	2	0	

A significant difference was seen between the technique used and the total number of surgeries. All patients who received a distant flap needed one surgery. On the other hand, patients with a history of Z plasty, local flap, or release contracture with skin graft needed multiple surgeries.

Patient satisfaction with the technique used

Significant differences were noted between the technique used and the outcome, patient satisfaction, functional status, and complications. Release contracture surgery was associated with a higher percentage of excellent functional outcomes and an increased patient satisfaction score (Table 3).

**Table 3:** Patient satisfaction with the technique used

Technique		Acellular dermal matrix	Distant flap	Z plasty with local flap	Release contracture	Other	P-values
Outcome	Flat	0	0	8	52	2	0
	Hypertrophic scar	0	2	5	32	1	
	Keloids	1	0	1	0	0	
Functional status	Excellent	0	0	1	8	0	0.01
	Good	0	2	5	33	2	
	Poor	1	0	0	4	1	
Patient satisfaction	3	0	0	0	1	1	0
	4	1	0	0	0	0	
	5	0	0	0	2	1	
	6	0	0	1	4	0	
	7	0	2	3	16	1	
	8	0	0	2	16	0	
	9	0	0	0	3	0	
	10	0	0	0	2	0	
Complication	Complete graft loss	0	0	0	1	0	0.002
	Partial graft loss	0	0	0	2	1	
	Infection	0	0	0	1	0	
	contracture	0	0	3	10	0	
	None	1	1	11	70	1	

Burn and technique characteristics for patients requiring further surgery

Patient's gender and smoking status did not affect the need for further surgeries significantly. Most patients with a past surgical (78.3%) and burn unit admission (65.2%) history required multiple surgeries. Patients with thorax and abdomen burns had higher odds of requiring more surgeries. Patients with distant flaps and additional procedures had higher odds of further procedures (Table 4).

**Table 4:** Burn and technique characteristics for the need of further surgeries p < 0.05. Ref.: Reference group. ^1 ^Including hands. ^2^ Including feet. ^3^ Reference groups are all other groups.

Need for further surgeries		No % (n = 77)	Yes % (n = 23)	Univariable odds ratio (95% CI)
Total		77	23	-
Gender	Female	42.3	43.5	1.049 (0.41-2.68)
	Male	57.1	56.5	Ref.
Smoking status	No	74.4	82.6	Ref.
	Yes	25.6	17.4	0.61 (0.185-2.01)
Patient received a previous surgery	No	65.4	21.5	Ref.
	Yes	34.6	78.3	6.8 (2.27-20.32)
Patient had been previously in a burn unit	No	67.9	34.8	Ref.
	Yes	32.1	65.2	3.97 (1.49-10.6)
Body location burned^3^	Upper limb^1^	62.8	60.9	0.92 (0.35-2.39)
	Lower limb^2^	34.6	21.7	0.52 (0.17-1.56)
	Thorax and abdomen	10.3	30.4	3.82 (1.21-12.09)
	Head and neck	21.8	13	0.53 (0.14-2.02)
The technique used in the present surgery^3^	Z plasty with local flaps	16.7	21.7	1.38 (0.43-4.41)
	Release contracture with a skin graft	93.6	87	0.45 (0.1-2.07)
	Acellular dermal matrix	0.00	4.3	0.22 (0.15-0.31)
	Distant flap	1.3	4.3	3.5 (0.21-58.25)
	Other surgery	1.3	8.7	7.33 (0.63-84.85)

## Discussion

Management of burn patients is a major burden in plastic surgery. In the past decade, significant improvement was achieved in acute resuscitation, wound management, sepsis treatment, and surgical management [7]. This advancement decreased the burning mortality and enhanced the post-burn functional outcomes [8]. Burn complications such as scarring and contractures can hamper functional capacity and be psychologically devastating for burned patients, especially children. This was the cornerstone of many previous studies that assessed functional outcomes and the independence level of patients post burn along with the physicians’ efforts in treating burns [9-12].

Various surgical methods are available for burn reconstruction with different advantages and disadvantages. These include skin grafts, local flaps, regional flaps, free flaps, prefabricated flaps, superthin flaps, and dermal scaffolds [12]. Besides that, noninvasive surgical methods are an option for scar management [7].

Multiple articles in the literature described various surgical procedures along with the most suitable technique with reference to the body area [13]. Skin grafts and locoregional flaps are the mainstays in burn reconstruction [14], and microsurgical techniques using free tissue transfer are a potential alternative [14,15].

Despite the heightened interest in reconstructive plastic surgery, there is a scarce amount of articles assessing the epidemiological characteristics of burned patients. Most of the patients who underwent reconstructive surgeries were operated on the upper limb. This is similar to a study done by Hop et al. where 57.4% of the reconstructive surgeries were carried out on the upper limb. They also stated that a burn in the upper limb is a risk factor for undergoing reconstructive surgery. Moreover, studies have shown a high prevalence of scar contractures after hand burns. A prevalence of 23% was identified by Schneider et al. [16] (seven patients in a study by van der Vlies et al. [17] and eight in a study by Zuo et al. [7]). These contractures will affect daily life activities and have a great impact on the quality of life [17]. This might be the cause behind the high percentage of patients seeking reconstructive surgery in their upper limbs.

Moreover, in our subgroup, most of the patients undergoing reconstructive surgery were initially burned by the flame, followed by scald burns. This is also similar to a study done by Hop et al. where a flame injury was considered a predictor of future reconstructive surgery. Similarly, the flame injury was considered a risk factor for reconstructive surgery in hand burn patients [17]. Multiple studies tried to assess the prevalence of scar contractures post-burn. While most studies considered the prevalence of contracture linked to the site of the burn, others considered age, cause of the burn, and gender as the main factors affecting the prevalence of contractures. In a systematic review, the predictors of future reconstructive surgeries were burns to the upper limb, flame burns, and a higher percentage of TBSA burns [18]. It is worth noting that the prevalence of scar contractures can be influenced by ethnicity. When we compared the number of previous surgeries in our sample to the patients admitted for surgery, we noticed that patients requiring multiple surgeries had an initial burn in the upper limb and maxillofacial area, had a flame burn injury initially, were younger, were previously admitted to the burn center, and were female patients. However, these values were not statistically significant due to the small sample size.

When comparing the surgical technique used in the first reconstructive surgery to the number of previous surgeries, 60% of the patients undergoing skin grafts at their first surgery underwent multiple interventions. These values were statistically significant. This means that skin grafting can increase the number of later surgeries. The defect in burn patients is due to skin loss, so skin grafts are the most common technique used in these patients [19]. The most common procedure used in our study was the release of contracture with skin grafting followed by release contracture and local flaps. This is similar to other studies where release contracture, skin grafting, and local flaps are the worship for burn reconstruction [12].

Moreover, patient satisfaction, aesthetic outcome, and low complication rates were observed with skin grafts. These values were statistically significant. In a study by Hop et al., the most common technique used in 10 years of follow-up was release with local flaps or skin grafting.

Limitations

We had a possible selection bias due to the method used in selecting the sample, which may not be representative of the Lebanese population; the participants included were from one center. However, this is the biggest burn center in Lebanon. Another limitation was due to the absence of group control; thus, our study was circumscribed to a form of pooled data.

## Conclusions

This is the first epidemiological study evaluating the medical and general characteristics of patients undergoing reconstructive surgeries for burn scar treatment in the Lebanese population. According to our study, the most common reconstructive surgery performed for burn scar treatment was contracture release coupled with skin grafting. The most frequent cause of burns among the Lebanese population was flame burn, which affected mainly the upper limb. A significant difference was seen between the different techniques used and the total number of surgeries. Further studies recruiting patients from all over Lebanon and assessment of their characteristics are now warranted.
